# Enhanced spatial analysis assessing the association between PFAS-contaminated water and cancer incidence: rationale, study design, and methods

**DOI:** 10.1186/s12885-025-13508-2

**Published:** 2025-01-17

**Authors:** Resa M. Jones, Erin R. Kulick, Ryan Snead, Robin Taylor Wilson, John Hughes, Ted Lillys

**Affiliations:** 1 Department of Epidemiology and Biostatistics, College of Public Health, Temple University, 1301 Cecil B. Moore Ave. Ritter Annex, 9th Floor, Rm 917, Philadelphia, PA 19122 United States; 2 Fox Chase Cancer Center, Temple University Health, Philadelphia, Pennsylvania United States; 3 Department of Community and Population Health, College of Health, Lehigh University, Bethlehem, Pennsylvania United States; 4 Research Triangle Institute, International, Cary, North Carolina United States

**Keywords:** Bayesian, Cancer incidence, Index model, PFAS, Residential history, Spatiotemporal, Drinking water

## Abstract

**Background:**

Cancer is a complex set of diseases, and many have decades-long lag times between possible exposure and diagnosis. Environmental exposures, such as per- and poly-fluoroalkyl substances (PFAS) and area-level risk factors (e.g., socioeconomic variables), vary for people over time and space. Evidence suggests PFAS exposure is associated with several cancers; however, studies to date have various limitations. Few studies have used rigorous spatiotemporal approaches, and, to our knowledge, none have assessed cumulative exposures given residential histories or incorporated chemical mixture modeling. Thus, spatiotemporal analysis using advanced statistical approaches, accounting for spatially structured and unstructured heterogeneity in risk, can be a highly informative strategy for addressing the potential health effects of PFAS exposure.

**Methods:**

Using population-based incident cancer cases and cancer-free controls in a 12-county area of southeastern Pennsylvania, we will apply Bayesian spatiotemporal analysis methods using historically reconstructed PFAS-contaminated water exposure given residential histories, and other potential cancer determinants over time. Bayesian group index models enable assessment of various mixtures of highly correlated PFAS chemical exposures incorporating mobility/residential history, and contextual factors to determine the association of PFAS-related exposures and cancer incidence.

**Discussion:**

The purpose of this paper is to describe the Enhanced PFAS Spatial Analysis study rationale, study design, and methods.

## Background

Evidence suggests exposure to per- and poly-fluoroalkyl substances (PFAS) may be associated with several cancers such as kidney, thyroid, testicular, ovarian, and non-Hodgkin lymphomas.^1–6^ PFAS are manufactured chemicals used for decades in firefighting foam and waterproofing materials, nonstick cookware, stain-resistant fabrics, and cosmetics.^7^ During production and use, PFAS transfers into the soil, water, and air where they remain as ‘forever chemicals’ due to their chemical properties. Exposure to these chemicals is ubiquitous and can occur in various ways such as ingestion, dermal contact, and inhalation of contaminated soil or air. The main source of exposure is drinking contaminated water; the United States (US) Geological Survey (USGS) estimates 45% of all US tap water is contaminated by ≥ 1 PFAS types.^8^

PFAS contamination levels are high in surface water and groundwater around current and former military bases such as those in Bucks and Montgomery Counties Pennsylvania (PA), which have measured PFAS levels in drinking water up to 1,290 parts per trillion (ppt) – significantly higher than the federal minimum reporting levels (MRL) of 4 ppt for PFOA and PFOS.^9–11^ Additionally, preliminary data of local adults has shown that PFAS blood concentration levels exceed what would be expected in about 95% of the US population.^8^

PA has the second highest age-adjusted cancer incidence rate (505.8/100,000) in the US.^12^ The multistep process of cancer development occurs slowly over time, 20–30 years for many cancers, with increasing capacity for cancer initiation, differentiation, and proliferation over the life course.^13–15^ Individual-level factors like age and genetics are well-studied significant, non-modifiable risk factors for cancer. However, most risk factors for cancer vary for people over time and space such as occupational and environmental exposures (e.g., PFAS), intrapersonal risk factors (e.g., obesity, socioeconomic status), and lifestyle behaviors (e.g., diet, physical activity). Additionally, recent research increasingly recognizes the vital role of area-level social and structural determinants of health (e.g., neighborhood-level socioeconomic status) in influencing cancer outcomes.^16–23^ Public health researchers generally embrace the concept that “place matters” when considering health and health equity, yet few studies to date capture varying patterns of geospatial and neighborhood exposures for individuals as they move throughout their life course.^24–27^ A substantial limitation of prior studies is the fact that mobility data as well as area-level context have not been included; thus, they are unable to capture varying patterns of geospatial and neighborhood exposures for individuals as they change residential locations over the life course. ^27^ Most studies use patient address at time of diagnosis as a proxy for lifetime exposures. However, this approach may substantially misclassify true exposure leading to biased estimates of the true risk of PFAS exposure related to cancer development. This is critical given ~ 10–20% of US adults moved annually from 1965 to 2012.^15^ Similarly, PA data suggest that 8.4-13.3% of residents in our 12-county area moved in a single year.^28^

We have the unique opportunity to link PFAS exposure data over time to cancer incidence in 12 counties in Southeastern PA, a wide geographic area with significant ranges of PFAS contamination. Additionally, we will develop residential histories for all participants, enhancing measurement of PFAS exposure, addressing limitations of previous studies that have not used spatial statistics.^27^

Rigorous spatiotemporal analytic methods are a highly informative strategy for addressing the potential health effects of pollutant exposures varying over time and locations. Few studies have used rigorous spatiotemporal approaches, and none have incorporated chemical mixture modeling. While a scientific standard to combine these exposures does not yet exist,^29–33^ the use of Bayesian group index models enables assessment of the effect of various mixtures of highly correlated PFAS chemical exposures incorporating mobility/residential history, and contextual factors to determine the association PFAS-related exposures and cancer incidence.^34–39^ These models estimate weights for each variable through an index and can detect important variables in the combination of variables with high sensitivity and specificity. Further, Bayesian models provide a flexible approach to modeling risk while accounting for spatially structured and unstructured heterogeneity in risk.

### Study objectives

To advance our understanding of the association between PFAS contamination and cancer incidence and address the limitations of research to date, we designed a population-based case-control study, the Enhanced Spatial Analysis Assessing the Association between PFAS-Contaminated Water and Cancer Incidence study (Enhanced PFAS Spatial Analysis). We will apply advanced Bayesian spatial analysis methods using historically reconstructed PFAS-contaminated water exposure given case and control residential histories, and other potential cancer determinants over time for multiple cancer endpoints in 12 Southeastern PA counties. The purpose of this paper is to describe the design and methods of the Enhanced PFAS Spatial Analysis study.

## Methods

### Study design and setting

**Fig. 1:**
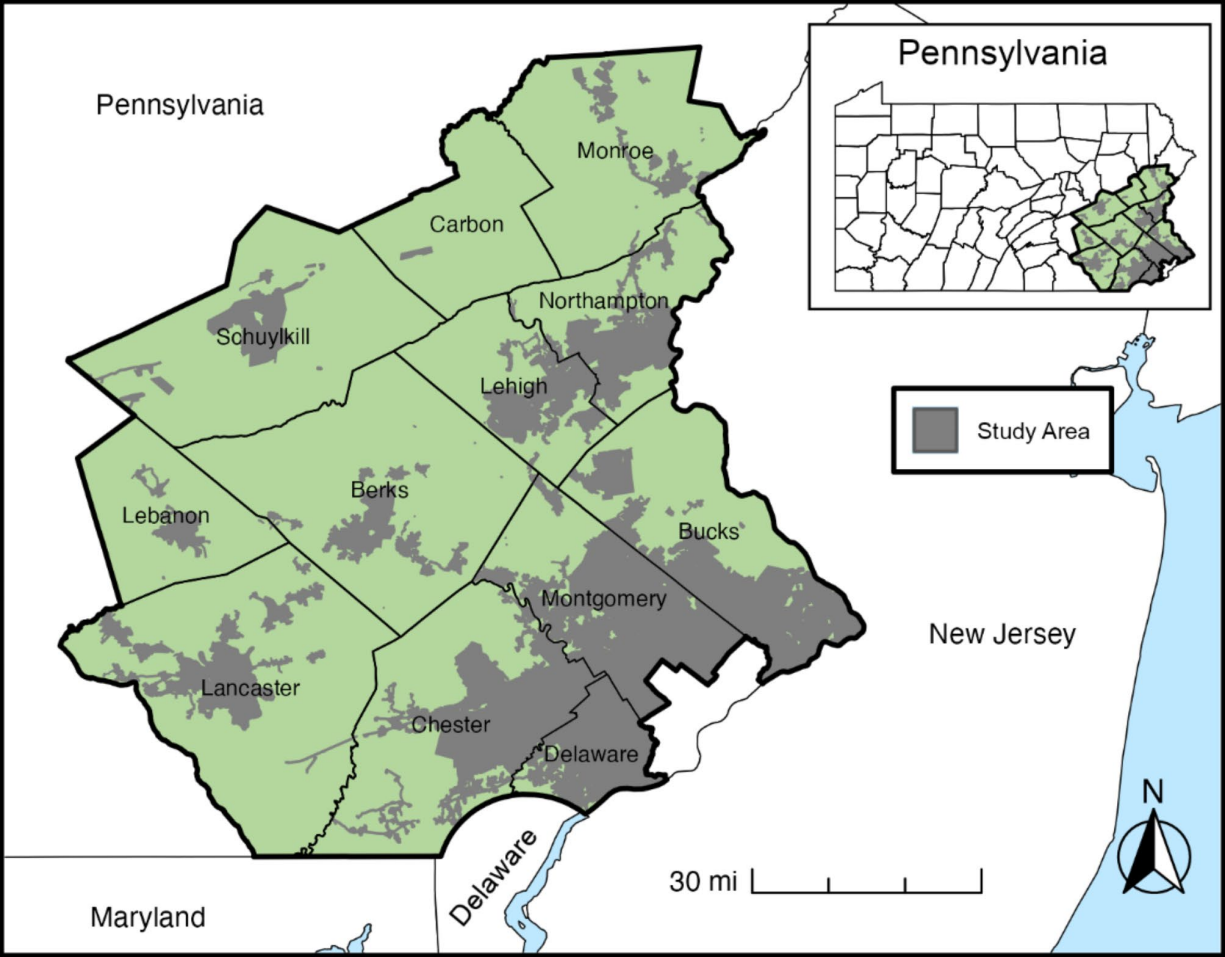
Study area for the enhanced spatial analysis assessing the association between PFAS-contaminated water and cancer incidence (Enhanced PFAS Spatial Analysis study)

The Enhanced PFAS Spatial Analysis study is a secondary data analysis of previously collected data using a frequency-matched case-control study design with adult (18 + years) population-based incident cancer cases and cancer-free controls living in 12 southeastern PA counties (i.e., Berks, Bucks, Carbon, Chester, Delaware, Lancaster, Lebanon, Lehigh, Monroe, Montgomery, Northampton, and Schuylkill). The geographic catchment areas with PFAS exposure data are shown in Fig. 1. Advanced spatial analyses will be used to assess the association between historical PFAS exposure, area-level social determinants, and cancer incidence. This project was reviewed and approved by the Temple University Institutional Review Board (#265091), which includes a waiver of consent (per 45 CFR 46.116 [d]) and a HIPAA waiver of authorization (per 45 CFR 164.512(i)(1)(ii)).All data will be kept confidential, and no results will be presented at the individual level, so it is impossible to identify any individual.

### Data sources and measures

#### Cancer registry data

The primary outcome is cancer incidence, with a focus on cancers that are most likely to be associated with PFAS exposure and other biologically plausible cancers: bladder, brain/central nervous system, breast (female), colorectal, endometrial (female), kidney, liver, non-Hodgkin lymphoma, ovarian (female), prostate (male), testicular (male), and thyroid. For the 12-county area, we will obtain incident cases diagnosed between 2000 and 2020 from the PA Cancer Registry, a population-based registry with high data completeness. [40, 41]

Histologically confirmed, invasive incident cancer cases will be eligible if they were ≥ 18 years, resided in the 12-county study area at the time of cancer diagnosis, and had at least one of 12 invasive cancer diagnoses from 2000 to 2020; there were 288,618 invasive incident cancer cases in the study catchment area (Table 1). Thus, the total study population will include ~ 866,000 people (~ 577,236 2:1 matched cancer-free controls plus cases). Using secure, encrypted data transfer procedures, we will obtain key variables, which can be classified into three broad categories – cancer incidence, address to obtain residential history and geocoding (see below), and demographics. We will obtain first and subsequent primary sites, date of diagnosis, stage at diagnosis, histology, and grade. We will only include malignant tumors (i.e., excluding benign and borderline). We will also obtain tumor size and “sequence numbers” (i.e., number of previously diagnosed primary cancers). We will ascertain residential address at time of diagnosis to ultimately assess the effect of area-level PFAS exposure on cancer incidence.

**Table 1:** Incident cancer cases of interest in the 12-County study area, 2000–2020

County	Primary Cancer Site											
	Bladder	Brain/CNS	Breast (female)	Colorectal	Endometrial (female)	Kidney	Liver	NHL	Ovarian (female)	Prostate (male)	Testicular (male)	Thyroid
Berks	189	501	4871	3662	1079	1228	531	1631	401	4759	213	938
Bucks	3921	1042	10,525	6818	2526	2458	1002	3127	1078	10,049	427	2373
Carbon	27	4	55	59	15	15	4	24	11	50	0	14
Chester	2347	724	7396	4254	1570	1464	589	1988	725	6612	307	1956
Delaware	3228	851	9617	6972	2223	2227	1093	2622	934	8770	303	1926
Lancaster	2369	697	6767	4723	1740	1413	584	2087	683	6019	270	1478
Lebanon	518	130	1247	993	393	331	134	392	124	1137	43	226
Lehigh	2098	555	5306	3720	1465	1402	597	1675	544	5145	162	1492
Monroe	685	203	1791	1258	490	407	196	503	204	1701	64	316
Montgomery	4692	1320	13,824	8502	3266	2911	1250	4083	1384	12,400	496	2472
Northampton	2138	469	4819	3396	1430	1323	555	1624	505	5021	194	1352
Schuylkill	628	137	1300	1266	390	352	144	457	151	1287	44	234
**Total**	**22,840**	**6633**	**67,518**	**45,623**	**16,587**	**15,531**	**6679**	**20,213**	**6744**	**62,950**	**2523**	**14,777**

Note: CNS: Central nervous system, NHL: Non-Hodgkin lymphoma

#### Population-based controls

To select cancer-free controls, we will obtain population-based lists from Marketing Systems Group,^42^ a multi-sourced database with information from publicly available and proprietary sources. Datasets will include name, current address, sex, and age. We will randomly select controls from census tracts with available PFAS exposure data and no cancer diagnosis. Two controls per case will be frequency matched by sex and 2-year age intervals given they were living in PA during the year of diagnosis for their corresponding case using a length of current residence filter.

#### Residential history and geocoding

We will buy publicly available and proprietary data from LexisNexis^®^^43^ for the residential history data (1970s-2020) for all cases and controls, which will enable us to create a variable for the assessment of historical PFAS exposure over space and time and (1) geocode all addresses and (2) generate a time-varying residential history. Previous studies using LexisNexis^®^ to analyze residential history data found a 90% match for city and state and 87% match for detailed addresses.^44^ We will use the same approach to create the residential history variable for each subject to model where people have lived since the 1970s and calculate their historic PFAS exposure and relevant area-level social and structural determinants of health to potentially identify etiologically relevant exposure periods. All addresses will be geocoded using ArcGIS Pro Streetmap Geocoder.^45^

#### PFAS-contaminated drinking water

Historical PFAS exposure reconstruction provides an important opportunity to validate novel spatial exposure-risk modeling. The reconstruction of time histories of PFAS-contaminated drinking water served by public water systems (PWS) will be based on primary and secondary data sources. Three primary data sources provide analytic results of PWS water samples: the Third and Fifth US Environmental Protection Agency’s (EPA) Unregulated Contaminant Monitoring Rule (UCMR) (i.e., UCMR3^46^ and UCMR5^47^) and PA Department of Environmental Protection (DEP)^48^ data for six PFAS chemicals (see below). Secondary data will come from a variety of sources such as locations where PFAS releases are known (e.g., superfund sites), highly suspected (e.g., firefighting training facilities, landfills, facilities manufacturing carpets, rugs, plastics, foils and coated paper bags, leather and tanning products, etc.) and suspected, information on community water source systems, and conditions collected under the auspices of the Safe Drinking Water Act (SDWA). Also, USGS data,^49^ the PA DEP’s GIS mapping tools,^50^ and the PA Hydrology Data Downloader^51^ will provide quantitative data to describe regional water table elevations, aquifer characteristics and extents, location and orientation of fractures, and other hydrologic and geologic data^52^ to help assess the direction and rate of groundwater flow. See details below.

#### Primary water data sources

##### EPA’s UCMR3 and UCMR5 data

The 1996 SDWA requires the EPA to test PWSs for unregulated contaminants at 5-year intervals to determine whether levels are sufficient to require adding them to the regulated contaminant list.^53^ The UCMR3 sampled six PFAS chemicals (i.e., PFBS, PFHpA, PFHxS, PFNA, PFOA, PFOS) in PWSs serving > 10,000 people and nationally representative PWSs serving < 10,000 people from January 2013 to December 2015.^46^ UCMR5 sampling began in 2023 and is currently ongoing on a rolling basis through 2026.^47^ UCMR5 includes 29 PFAS contaminants – the original six types measured in UCMR3 and 23 new subtypes.^54^ For historical exposure reconstruction, only the six subtypes in both UCMR3 and UCMR5 will be used. See Table 2 for EPA’s MRL for the six PFAS chemicals by UCMR3 and UCMR5.

**Table 2:** Environmental Protection Agency PFAS Minimum Reporting Level (MRL) in drinking water: UCMR 3 and UCMR 5

	Chemical					
	PFBS	PFHpA	PFHxS	PFNA	PFOA	PFOS
UCMR 3	0.09 µg/L	0.01 µg/L	0.03 µg/L	0.02 µg/L	0.02 µg/L	0.04 µg/L
UCMR 5	0.003 µg/L	0.003 µg/L	0.003 µg/L	0.004 µg/L	0.004 µg/L	0.004 µg/L

Note: EPA: Environmental Protection Agency; UCMR 3: Third Unregulated Contaminant Monitoring Rule; UCMR 5: Fifth Unregulated Contaminant Monitoring Rule Data MRL: Minimum Reporting Level; PFBS: perfluorobutanesulfonic acid; PFHpA: perfluoroheptanoic acid; PFHxS: perfluorohexanesulfonic acid; PFNA: perfluorononanoic acid; PFOS: perfluorooctanesulfonic acid; PFOA: perfluorooctanoic acid

##### Pennsylvania Department of Environmental Protection (PA DEP)

The PA DEP performed additional PFAS sampling from May 2019 to March 2021 in PWSs with high potential for contamination due to proximity to common sources of PFAS for all six PFAS chemical in UCMR3 and UCMR5.^48^

#### Secondary water data sources and measures

Secondary water data sources fall into two categories: sources with information on location of known or suspected releases of PFAS into the environment that can migrate to groundwater, and sources that help determine the likely direction of PFAS transport if they were to reach the groundwater. The secondary sources described below will be used to identify study area locations where PFAS-containing materials are known, highly likely, or suspected to be stored and used or where wastes are generated as part of the normal activities. The proximity of these locations to PWSs and their operational history, if known, influence the assignment and slope of estimated historical concentrations in water provided by the PWS.

##### Location-based sources

###### EPA’s superfund sites

The EPA cleans the most polluted land (i.e., superfund sites) in the US, including known locations of PFAS discharge and contamination. The locations of superfund sites are publicly accessible.^55^

###### Pennsylvania Department of Environmental Protection (DA DEP)

The PA DEP collected and mapped firefighter training sites as part of their PFAS sampling plan,^48^ which includes longitude and latitude plotting point data. Additionally, location data for residual (i.e., industrial, mining, or wastewater treatment facility) and municipal waste operations (e.g., landfills) are accessible via PA’s open-access geospatial data portal.^49^ Firefighter training sites and waste operations represent locations where PFAS-containing materials like aqueous film-forming foams, biosolids, and other solid wastes are managed or released directly into the environment.

###### EnviroFacts‘ facility registry service

A publicly available database of all facilities under environmental regulation or interest is available.^56^ Data contains key facility information on manufacturing sites in PA, including location and standard industrial classification (SIC) codes. Table 3 provides SIC codes representing industries that are very likely to generate PFAS-containing solid or liquid wastes on site [57, 58]. Regulated study area facilities with these SIC codes will be considered as locations where PFAS-containing wastes are potentially released to the environment and their proximity to PWSs will be considered in assigning historic concentration trends to nearby PWS. SIC codes for industries that potentially generate solid or liquid PFAS-containing waste will also be considered.

**Table 3:** Standard industrial classification codes for industries very likely to generate PFAS-containing waste

Standard Industrial Classification Code	Industry
2262	Finishers of Broadwoven Fabrics of Manmade Fiber and Silk
2295	Coated Fabrics, Not Rubberized
2297	Non-woven Fabrics
2841	Soaps and Other Detergents, Except Specialty Cleaners
2842	Specialty Cleaning, Polishing, and Sanitation Preparations
3111	Leather Tanning and Finishing
3471	Electroplating, Plating, Polishing, Anodizing, and Coloring

###### Federal Aviation Administration (FAA)

The FAA’s Aeronautical Information Services data includes the geospatial data for all regulated PA airports, which represent locations where aqueous film-forming foam releases may have occurred during either fire training exercises or during air traffic accidents.^59^

##### Flow-based sources

The following secondary sources will be used to understand the likely groundwater flow directions that could transport PFAS to PWSs.

###### National Hydrography Dataset (NHD)

The NHD geodatabase provides the US water drainage network, catchment boundaries, and related surface water flow directions.^60^ Flow directions are indicators of the general surface slope of the drained region, which can establish general surficial aquifer flow direction within catchments and watersheds.

###### National Water Information System (NWIS)

The USGS NWIS repository and the Philadelphia Area Groundwater Level Network will provide historic groundwater elevation measurements, which will be used to create a series of seasonal groundwater elevation maps to further establish groundwater flow directions.^61,62^

###### Geologic maps of Pennsylvania

The PA Department of Conservation and Natural Resources maintains a geodatabase of geologic maps including bedrock, surficial geology, and faults, which will be used to qualify underlying assumptions regarding groundwater flow direction obtained from other sources, identify preferential pathways (e.g., fracture flow) for PFAS in groundwater.^63^ In overburden aquifers or aquifers composed of sediment (e.g., sand, gravel, weathered rock), groundwater flow generally follows the slope of ground surface elevation. Fractured sedimentary rock aquifers (e.g., Lockatong and Stockton Formations) in the study area can have preferential pathways for flow.^64^

### Historical reconstruction of PFAS exposure

Historical PFAS concentration trends at each PWS will be derived in stages, beginning with all primary data collected from PWSs. A best-fit linear line using all available analytical data for each of the six PFAS will be constructed and where possible (8 data points or more), the Theil-Sen method will be applied to estimate trend and slope.^65^ The shape and slope of individual hindcasts will be conditioned using nearby secondary data as available. Secondary data will be incorporated and evaluated in a structured manner using methods derived from the field of multi-criteria decision analysis: Multi-Attribute Utility Theory (MAUT).^66^ MAUT provides a straightforward, arithmetic approach to account for uncertainty in the identified likeliest trend of historic drinking water concentrations and how primary and secondary data are evaluated. Secondary data sets for each PWS will be individually evaluated to develop a utility/influence function to quantify the impact on the characteristics of the interpolated trend. We will model the strength of the trend (e.g., are we observing a steady state process), the trend slope or shape (e.g., are observations from rising or falling PFAS concentrations), and the mix of water sources (e.g., groundwater vs. surface water).

NHD flow lines^57,61^ provide a convenient basis for tracing the likely path of groundwater flow direction through successively larger areas from a catchment that contains a known or suspect source of PFAS “downhill” in a search for intersections with PWS or well fields that serve a PWS (if known).

Regional groundwater elevation or potentiometric surface maps will be developed using USGS NWIS^67^ and Philadelphia Area Groundwater Level Network^62^ data. Groundwater flow directions can be derived from groundwater elevation contours by drawing lines perpendicular to elevation contours^68^ and will be used in conjunction with additional sources of flow direction information to support the development of flow pathways from known or suspected sources to PWS.^51,64^ If pathways can be established between a source and area where groundwater is used for drinking water, the nature of source (e.g., firefighting training ground, or manufacturing facility), its age (if known), and the distance of travel all can be used to condition or weight the likelihood that PFAS concentrations have been increasing, decreasing, or stable with respect to current measurements.^66^

Finally, using the reconstructed PFAS exposure and the residential history for each case and control, cumulative exposure to each of the six PFAS subtypes will be estimated over time incorporating uncertainty as appropriate.

### Area-level SES data

To estimate time-varying area-level socioeconomic measures at the census tract and block group, we will obtain US Census Bureau decennial data (1980, 1990, 2000, 2010, 2020) and 5-year estimated data from the American Community Survey starting in 2005.^69^ Many of these variables are based on our previous work on neighborhood deprivation and colorectal cancer screening adherence^70^ and lead exposure.^37,71^ Additional social determinants and SES-related variables may be included given the conceptual framework for the specific cancer-related outcome being assessed. The area-level SES variables will be linked to cases and controls given their residential histories (Table 4).

**Table 4:** Example SES area-level candidate variables from the US Census Bureau and the American Community Survey

Median household income	% Veterans
Per capita income	% population 1 + years in same house
Gini index	Average household size
% household with public assistance	% vacant housing units
% household with social security income	% housing units with > 1.5 person per room
% families with children < 18 years in poverty	% renter-occupied households
% population in poverty	Median rent
% households in poverty	% owner-occupied households with mortgage
% female	% housing units with no telephone service
% male	% housing units with no vehicle available
% Black	% housing units with no heating fuel
% Hispanic	% housing units lacking complete plumbing
% foreign-born	% housing unit lacking complete kitchen facilities
% who speak a foreign language	% population 25 + years with bachelor’s degree

### Statistical analysis

Bayesian index models will be used to determine the association between PFAS-related exposures and cancer incidence. This mixture modeling approach will allow us to assess the effect of exposure to a relatively large number of correlated PFAS components while also considering contextual factors (e.g., socioeconomic variables), residential history, and spatial correlation in disease risk. These models estimate weights for each component in a weighted index and can detect important components in the index with high sensitivity and specificity. We will model the probability of cancers because we have population-based cancer cases and controls. We will reconstruct historical PFAS exposure and specify a PFAS contamination index for each subject based on census tract or block group of residence using a weighted combination of the quantiles of the PFAS chemicals where the weights are given a Dirichlet prior with parameters. The weight $\:{\omega\:}_{j}$ represents the relative importance of the$j{\text{th}}$PFAS variable in the index. We will use the following model:

$$\begin{array}{l}{\mathop{\rm logit}\nolimits} \left( {{p_i}} \right) \\= {\beta _0} + {\beta _1}\left( {\sum\limits_{j = 1}^C {{\omega _j}{q_{ij}}} } \right) + {\beta _2}{z_i} + \sum\limits_{k = 1}^m {{\gamma _k}{b_{ik}} + {u_i}}\end{array}$$

where${\beta _0}$is the intercept, ${\beta _1}$is the effect for the index,${\beta _2}$is the coefficient for age${z_i}$of the *i*^th^ subject, $\:{b}_{ik}$ are spatially structured basis functions, $\:{\gamma\:}_{k}$ are basis coefficients, and $\:{u}_{i}$ is an unstructured random effect. Our use of a basis expansion to model spatial dependence, known as fixed rank kriging,^72,73^ will permit us to flexibly and efficiently accommodate extra spatial structure.

The spatial effects can be included at the individual-level based on residential location or area-level (e.g., census tract).^74^ The spatial basis functions can be multiresolution bisquare or radial basis functions computed on a mesh laid over the study region such that the mesh has a higher density where the data density is higher, a lower density where the data density is lower.^74^ A number of sensible prior distributions for the basis coefficients are available. The unstructured random effects can follow independent Gaussian distributions. We will identify areas as being significantly elevated for cancer risk using posterior estimates of exceedance probabilities.

In addition, we will use Bayesian group index models to assess PFAS exposures and neighborhood characteristics with multiple indices for different times based on residential histories. The Bayesian group index model includes a neighborhood characteristics index and associated effect as well as PFAS exposure estimates for multiple times of interest as:

$$\begin{array}{l}\log {\rm{it}}({p_i})\\= {\beta _0} + \sum\limits_{t = 1}^T {{\beta _{1t}}\left( {\sum\limits_{j = 1}^T {{\omega _{jt}}{q_{ijt}}} } \right) + } {\beta _2}\left( {\sum\limits_{t = 1}^T {{\omega _t}{r_{it}}} } \right)\\+ z_i^\prime \phi + \sum\limits_{k = 1}^m {{\gamma _k}{b_{ik}} + {u_i}} \end{array}$$

where $\:{\beta\:}_{1t}$ is the PFAS exposure at different times, $\:{\beta\:}_{2}$ is the cumulative area-level characteristic measure, and $\:{\omega\:}_{t}$ is the weight of the area-level characteristics at time *t*. This model includes the weighted index of PFAS exposure over time to handle correlation between the measures while still allowing estimation of the importance weights for them. The set of variables defining PFAS exposures and area-level characteristics do not change over time, but the weights for the variables in the indices and index effects $\:{\beta\:}_{1t}$ do change. Thus, the importance of individual PFAS types and area-level variables can change over time as well as the index. This model can be used to identify the most important timing of exposures among the set of times considered through posterior inference on the index effects $\:{\beta\:}_{1t}$ and the most important variables in each index through posterior inference on the weights $\:{\omega\:}_{jt}$.

We will use Stan^75^ and R^76^ to do posterior inference for the index models and the group index models. Stan provides a flexible probabilistic programming language that can be used to program the models. Stan employs a Hamiltonian Monte Carlo sampling algorithm, which offers substantial advantages over competing approaches.

### Power

In a simulation based on SEER non-Hodgkin lymphoma data where we set 5 of 27 available chemicals (3 PCBs, 1 PAH, 1 pesticide) to be associated with the outcome, we found the power of detecting a significant mixture effect exceeded 90% for *N* ≥ 50. Power, sensitivity, and specificity were > 90% for *N* ≥ 200.

## Discussion

We have designed a novel population-based case-control study called the Enhanced Spatial Analysis Assessing the Association between PFAS-Contaminated Water and Cancer Incidence study (Enhanced PFAS Spatial Analysis) to advance the understanding of the association of PFAS-contaminated water and cancer incidence using historical reconstruction of PFAS exposure and robust spatiotemporal modeling.

The strengths of the Enhanced PFAS Spatial Analysis study address key methodological deficiencies in the current literature.^1^ First, many cancers have decades-long lag times (e.g. 20 + years) between exposure and subsequent diagnosis and cumulative environmental/socioeconomic exposures related to cancer risk are inherently spatial and temporal because people move frequently throughout life.^13–15^ Annually 10–20% of the US population moves, which can vary by socioeconomic and demographic factors.^15^ Existing studies infrequently account for the mobility of subjects over the life course and instead use the patient address at time of diagnoses or a single blood measurement as a proxy for a lifetime of exposures,^27^ which results in measurement error and misclassification of exposure that may result in biased risk estimates. Further, such studies have diminished power to detect a significant historic or cumulative effect especially for disease outcomes with long latency, like cancer. The Enhanced PFAS Spatial Analysis study will reconstruct residential histories for both cases and controls over decades, giving us the ability to reconstruct historic environmental exposures for participants over time. To our knowledge, the Enhanced PFAS-SA project will be the first study to use public record database-generated residential histories over 40 years to estimate the effects of historic PFAS exposure and area-level sociodemographic characteristics. Second, a key strength of the exposure modeling approach is that it is relatively simple, straightforward, and conservative in that it is likely to overestimate the concentration-time histories. Additionally, it uses as much measured, publicly available data to support calculations as possible, and minimizes the use of assumptions to apply qualitative data in developing hindcasts of drinking water concentration. A third strength of the Enhanced PFAS Spatial Analysis study is that it uses random population-based sampling to identify cancer-free controls, an improvement over previous studies that have used other cancer cases as control, which may bias effects toward the null due to shared risk factors across cancers. Lastly, our novel statistical and spatiotemporal methods address many weaknesses of prior studies. Earlier studies did not consider any spatial dependence in risk, an inherent characteristic of environmental exposures. Additionally, much of the research on environmental pollutants to date has examined single chemicals without a more holistic approach to highly correlated mixtures of PFAS (e.g., PFOA, PFOS, PFBS, PFNpA, PFHxS, and PFNA) in the environment, which biases estimates. Bayesian index models will overcome methodological limitations of prior approaches while also providing easily interpretable estimates of association. These methods will permit consideration of the context and effect of time and place on cancer along with highly correlated PFAS types and can provide a more inclusive view of the environmental and neighborhood context.

A few limitations should be noted. Due to sampling costs and the large geographic area, this study will rely on PFAS sampling conducted by the EPA and PA DEP in PWSs and does not include about 22% of the catchment area population with private wells.^77^ Also, there are limited analytical data points over 10 years to develop time histories for each PWS (range: 1–19). In addition, heterogeneities of drinking water concentration in PWSs cannot be completely estimated without detailed plans and operations these systems. Assuming that the hydrogeology is primarily composed of overburden, as opposed to fractured flow, will lead to an overestimation in the assumed spatial distribution of contaminants in groundwater. However, the study region is highly fractured and depending on the size and orientation of fractures, groundwater can move substantially faster, and result is less dilution and dispersion of dissolved contaminants.^64^

In summary, we are using a methodologically complex historical reconstruction strategy to estimate PFAS exposure over time using publicly available PWS data, a method that has not been used previously in PFAS studies.

## Data Availability

No datasets were generated or analysed during the current study.

## Declarations

### Disclaimer

The protocol and information in this manuscript are those of the authors.

## Abbreviations

DEP: Department of Environmental Protection
EPA: Environmental Protection Agency
FAA: Federal Aviation Administration
MRL: Minimum reporting levels
NHD: National Hydrography Dataset
NWIS: National Water Information System
PA: Pennsylvania
PFAS: Per- and poly-fluoroalkyl substances
PFBS: Perfluorobutanesulfonic acid
PFHpA: Perfluoroheptanoic acid
PFHxS: Perfluorohexanesulfonic acid
PFNA: Perfluorononanoic acid
PFOS: Perfluorooctanesulfonic acid
PFOA: Perfluorooctanoic acid
PWS: Public water systems
SWDA: Safe Water Drinking Act
UCMR: Unregulated Contaminant Monitoring Rule
